# The Effectiveness of Acupuncture in Management of Functional Constipation: A Systematic Review and Meta-Analysis

**DOI:** 10.1155/2020/6137450

**Published:** 2020-06-17

**Authors:** Lu Wang, Mingmin Xu, Qianhua Zheng, Wei Zhang, Ying Li

**Affiliations:** ^1^School of Acupuncture–Moxibustion and Tuina, Chengdu University of Traditional Chinese Medicine, Chengdu 610075, China; ^2^Office of Educational Administration, Chengdu University of Traditional Chinese Medicine, Chengdu 610075, China; ^3^Graduate School, Chengdu University of Traditional Chinese Medicine, Chengdu 610075, China

## Abstract

**Objective:**

The purpose of this study was to assess the effectiveness and safety of acupuncture for functional constipation (FC).

**Methods:**

A rigorous literature search was performed in English (PubMed, Web of Science, the Cochrane Library, and EMBASE) and Chinese (China National Knowledge Infrastructure (CNKI), Chinese Biological Medical (CBM), Wanfang database, and China Science and Technology Journal (VIP)) electronic databases from their inception to October 2019. Included randomized controlled trials (RCTs) compared acupuncture therapy with sham acupuncture or pharmacological therapies. The outcome measures were evaluated, including the primary outcome of complete spontaneous bowel movement (CSBM) and secondary outcomes of Bristol Stool Form Scale (BSFS), constipation symptoms scores (CSS), responder rate, the Patient Assessment of Constipation Quality of Life (PAC-QOL) questionnaire, and safety evaluation. Meta-analysis was performed by using RevMan5.3.

**Results:**

The merged data of 28 RCTs with 3525 participants indicated that acupuncture may be efficient for FC by increasing CSBMs (*p* < 0.00001; MD = 0.84 [95% CI, 0.65 to 1.03]; *I*^2^ = 0%) and improving constipation symptoms (*p*=0.03; SMD = −0.4 [95% CI, −0.78 to −0.03]; *I*^2^ = 74%), stool formation (*p* < 0.00001; MD = 0.24 [95% CI, 0.15 to 0.34]; *I*^2^ = 0%), quality of life (*p* < 0.00001; *N* = 1, MD = −0.33 [95% CI, −0.45 to −0.21]), and responder rates (*p*=0.02; RR = 2.16; [95% CI, 1.1 to 4.24]; *I*^2^ = 69%) compared with the effects of sham treatment. No increased risk of adverse events was observed (*p*=0.44; RR = 1.18; [95% CI, 0.77 to 1.81]; *I*^2^ = 0%). With regard to medication comparisons, the pooled data indicated that acupuncture was more effective in increasing CSBMs (*p*=0.004; MD = 0.53 [95% CI, 0.17 to 0.88]; *I*^2^ = 88%) and improving patients' quality of life (*p* < 0.00001; SMD = −0.73 [95% CI, −1.02 to −0.44]; *I*^2^ = 64%), with high heterogeneity. However, there were no significant differences in responder rate (*p*=0.12; RR = 1.31; [95% CI, 0.94 to 1.82]; *I*^2^ = 53%), BSFS (*p*=0.5; MD = 0.17 [95% CI, −0.33 to 0.68]; *I*^2^ = 93%), or CSS (*p*=0.05; SMD = −0.62 [95% CI, −1.23 to −0.01]; *I*^2^ = 89%). Regarding safety evaluation, acupuncture was safer than medications (*p* < 0.0001; RR = 0.3; [95% CI, 0.18 to 0.52]; *I*^2^ = 30%).

**Conclusions:**

Current evidence suggests that acupuncture is an efficient and safe treatment for FC. Acupuncture increased stool frequency, improved stool formation, alleviated constipation symptoms, and improved quality of life. However, the evidence quality was relatively low and the relationship between acupuncture and drugs is not clear. More high-quality trials are recommended in the future. PROSPERO registration number: CRD42019143347.

## 1. Introduction

Functional constipation (FC) is one of the common functional bowel disorders that affect approximately 14% of the adult population worldwide [1]. One survey study indicated that the most frequent symptoms of FC were decreased defecation frequency, difficult stools, feelings of incomplete evacuation, and abdominal discomfort [2]. Although FC is not life-threatening, it has a very significant adverse impact on quality of life and increases economic costs [3, 4]. Risk factors for FC include female sex, older age, and reduced caloric intake [5, 6]. These adverse effects make the management of constipation a major clinical issue.

Many guidelines and reviews summarize stepwise clinical therapeutic approaches from appropriate lifestyle and dietary modifications to various drug administration, including osmotic agents, stimulant laxatives, prosecretory agents, serotonin (5-HT4) receptor agonists, and probiotics, and so on [7, 8]. Anorectal biofeedback, nerve stimulation, and colonic surgery may be used to treat FC [9–11]. Although there are many methods to choose from, the side effects of these methods are notable, including diarrhea, bloating, nausea, and possible cardiovascular adverse events [12–14]. As a result, many people, including those who do not improve with existing medications or suffer many side effects, are interested in complementary alternative medicine.

According to a 2015 study, acupuncture and electroacupuncture were the most commonly used complementary and alternative therapies for constipation, followed by herbal medicine [15]. Acupuncture is an ancient Chinese medicine method in which acupuncture points on the skin are manually stimulated with needles. Acupuncture treats FC via regulation of the nervous system and peripheral gastrointestinal hormone contents [16, 17]. However, the current systematic review remained an uncertain conclusion whether acupuncture was effective in managing FC because of the miscellaneous outcome measures and diagnostic criteria and lack of high-quality repeatable multicenter randomized controlled trials (RCTs) [18]. Therefore, we performed a systematic review to evaluate the effectiveness and safety of acupuncture in the treatment of patients with FC via unification of measurement outcomes and inclusion criteria and the inclusion of high-quality RCTs.

## 2. Methods

This systematic review was registered in the PROSPERO registry (CRD42019143347), and the protocol was described previously [19]. The PRISMA guidelines and the recommendations of the Cochrane Handbook for Systematic Reviews of Interventions were complied with this systematic review and meta-analysis (Table S1) [20, 21].

### 2.1. Search Strategy

Two reviewers (WZ and QHZ) searched the databases from inception to October 2019, including four English databases (the PubMed, Web of Science, Cochrane Library, and EMBASE) and four Chinese databases (China National Knowledge Infrastructure (CNKI), Chinese Biological Medical (CBM), China Science and Technology Journal (VIP), and Wanfang Data Chinese databases). We used the following terms: (1) “acupuncture,” “manual acupuncture,” “electroacupuncture,” “acupuncture therapy,” or “acupuncture points,” combined with (2) “constipation,” “functional constipation,” “colonic inertia,” “dyschezia,” “astriction,” “obstipation,” or “slow transit constipation.” (See Table 1, for the search terms and strategy.) Because of the language restriction of our researchers, only studies published in English and Chinese were included.

### 2.2. Study Selection

#### 2.2.1. Inclusion Criteria

1. Participants: Patients over the age of 18 years who were diagnosed with FC using guidelines or the Rome IV/III/II criteria, regardless of demographic characteristics (ethnicity, comorbidity, gender, age) and severity of disease were included.
2. Study design: The trials were RCTs that used a two-, three-, or four-arm parallel design regardless of blinding.
3. Types of interventions: The intervention group was treated with acupuncture or electroacupuncture (EA), regardless of the number of acupuncture points, frequency, and courses of treatment. The control groups received no treatment, placebo acupuncture, sham acupuncture (SA), conventional medication, or placebo control.
4. Outcome measures: We limited the outcome measures to complete spontaneous bowel movement (CSBM), Bristol Stool Form Scale (BSFS), responder rate, constipation symptoms scores (CSS), the Patient Assessment of Constipation Quality of Life (PAC-QOL) questionnaire, and safety evaluation.

#### 2.2.2. Exclusion Criteria

1. Crossover trials, uncontrolled trials, quasi-randomized trials, reviews, case reports, and animal experimental research studies were excluded.
2. Studies with participants that included special populations, such as pregnant women, lactating women, or those diagnosed with constipation due to irritable bowel syndrome, were excluded.
3. We excluded trials in which the controls received acupuncture in combination with other methods, such as moxibustion, herbs, or medication and conventional medications that were not Western medicine, such as Chinese medicine, Tibetan medicine, and Zhuang medicine.
4. Trials that compared different points or forms of acupuncture were also excluded.
5. We excluded low-quality trials that had a clear risk of bias, such as a lack of randomized methods and incomplete data.
6. Duplicate publications and studies with incomplete data were also excluded.

### 2.3. Data Extraction and Quality Assessment

Two of the authors (MMX and LW) reviewed all titles and abstracts independently to determine the eligibility of articles. Argument between the two reviewers was solved via discussion and arbitration by a third reviewer (YL). The two authors made a final judgment by reading the full text of the remaining articles. A standardized data extraction form was used to extract detailed data from each selected study. The extraction information was collected according to a fixed protocol: study sites, total numbers, numbers of acupuncture and control participants, mean age, mean constipation duration, treatment duration, and outcomes. Missing information about the included trials was obtained by contacting the correspondent authors via e-mail.

The Cochrane risk of bias tool was used to assess bias in each study included by the two reviewers (LW and WZ). The risk of bias domains included random sequence generation, allocation concealment, blinding of participants and personnel, blinding of outcome assessment, incomplete outcome data, selective reporting, and other bias. The risk of bias in each domain was rated as “low,” “high,” or “unclear.” Disagreements were resolved via consultation with the third reviewer (YL). Finally, we evaluated the quality of evidence for the outcomes (acupuncture vs. SA) of the included studies in our review using the Grading of Recommendations Assessment, Development, and Evaluation (GRADE) guidelines [22].

### 2.4. Outcome Assessment

The primary outcome was CSBM. Secondary outcomes were BSFS, CSS, responder rate, PAC-QOL, and safety evaluation. The time point of all results was after treatment. A CSBM was defined as a bowel movement with the sense of complete evacuation that occurred without the use of any medication or other methods to assist defecation in the previous 24 hours. The BSFS is a seven‐hierarchy scale, with scores of 1–2 indicating constipation, 3–5 indicating normal stool, and 6–7 indicating diarrhea. The CSS assessed patients' eight constipation-related symptoms, including straining, endless sensation of defecation, bowel sound, abdominal pain, abdominal bloating, stool consistency, diarrhea, and fecal incontinence [23]. Responder rate was defined as the number of responders having at least three CSBMs per week divided by the total number of participants in each group. The PAC-QOL scored the effects of constipation on physical discomfort, psychosocial discomfort, anxiety, concerns, and satisfaction in their daily lives [24]. Higher scores indicated more defects or dissatisfaction. Safety evaluation was assessed using the adverse event reported in the studies.

### 2.5. Data Analysis

The Review Manager software program (version 5.3) was used for data synthesis. For continuous variables, such as CSBM, BSFS, CSS, and PAC-QOL, the mean difference (MD) or standard mean difference (SWD) with 95% confidence interval (CI) was used for analysis. For dichotomous data, such as the rates of responders and adverse events, the relative risk (RR) with 95% CI was utilized for analysis. Some studies reported change‐from‐baseline values instead of after‐treatment values. We calculated the after‐treatment values, assuming a correlation coefficient of 0.4 between baseline values and after‐treatment values according to the Cochrane handbook [25]. For studies that satisfied the predefined inclusion criteria with multiple intervention groups, if the multiple intervention groups used different acupuncture methods, we merged the data into a unified acupuncture group data. If the multiple intervention groups were different comparison groups, we implemented pairwise comparisons. For missing data, we contacted the corresponding authors via e-mail, otherwise the results were excluded. The magnitude of heterogeneity was measured using the *I*^2^ statistic: when *I*^2^ < 50%, a fixed-effects model will be used for pooled data; and when *I*^2^ ≥ 50%, a random-effects model was used. For each merged analysis, a heterogeneity test was performed using the chi-squared statistic. If *I*^2^ ≥ 50%, the synthesized studies were considered an indicator of a substantial level of heterogeneity. Subgroup or sensitivity analysis was performed to identify the cause. Subgroup analyses identified the possible factors that contributed to the heterogeneity, such as different acupuncture stimulation parameters, different control groups, participants' age, or disease course. And we evaluated publication bias by using funnel plots (*n* > 10).

## 3. Results

### 3.1. Search Results

**Figure 1:**
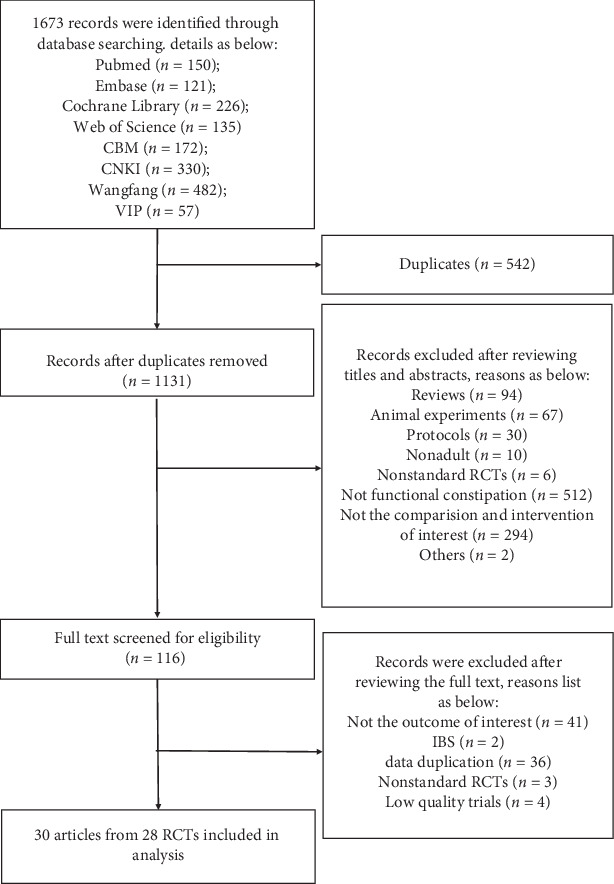
Flow diagram of the selection process.

According to the search strategy, a total of 1673 articles were identified. After duplicates were removed, 1131 articles were further evaluated using the eligibility criteria. Then, 116 articles were eligible for full-text evaluation after screening the titles and abstracts. We also excluded 86 articles for the following reasons: including IBS patients, no interested outcome indicator, repeated published data, not RCT, and low quality. Eventually, we included 30 studies in our system review [26–55]. Although 30 articles were included after screening, actually only 28 related RCTs (3525 participants) were extracted because data of 4 articles were from the same two RCTs (Peng, 2013; Mao, 2017 (2)), respectively [39, 40, 49, 50]. After reading the full text and analyzing the time period of study, we found that the outcomes from Mao, 2017 (2) were selectively reported in 2016 and 2017 separately. The same selective reporting is the RCT of Peng, 2013. The search process was showed in Figure 1.

### 3.2. Characteristics of the Studies

The included studies came from Korea and China and were published between 2010 and 2019. The diagnostic criteria of one RCT were the guidelines for clinical research [44], and the other RCTs were Rome III.There was 1 four-arm RCT [55], 5 three-arm RCTs [49–54], and 23 two-arm RCTs [26–48]. The treatment duration was set for 2 weeks in 2 studies [32, 46], 3 weeks in 1 study [47], 4 weeks in 18 studies [27, 30, 31, 33–37, 42, 44, 45, 48–55], and 8 weeks in 7 studies[26, 28, 29, 38–41, 43]. For these 28 trials, 10 trials reported CSBM [26–28, 36, 38–43, 46], 13 trials reported BSFS [27–29, 34, 35, 38–43, 47, 48, 53], 9 trials presented responder rate [26, 28, 29, 38–41, 43, 47, 53], 6 trials presented CSS [30, 45, 48–52], 10 trials mentioned PAC-QOL [28, 32, 33, 37, 38, 41, 43, 44, 46, 53], and 15 trials mentioned safety evaluation [26–33, 49–55].  Table 2 summarizes the other parameters of the included trials.

### 3.3. Risk of Bias Assessment

**Figure 2:**
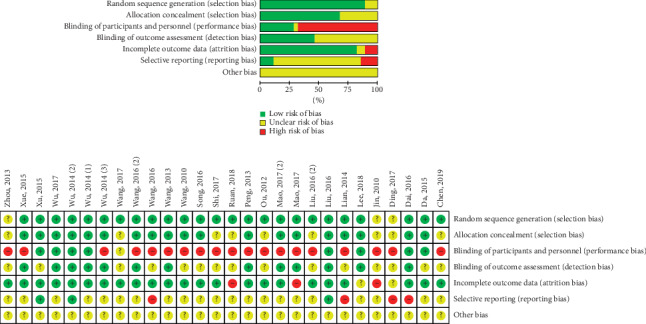
Risk of bias assessment.

Figure 2 summarizes the risk of bias in the 28 RCTs. Blinding of participants and personnel and incomplete outcome data may be the major reasons for selection bias and performance bias. Many studies were associated with an unclear risk of bias for blinding of outcome assessment, selective reporting, and other possible bias.

### 3.4. Acupuncture vs SA

**Figure 3:**
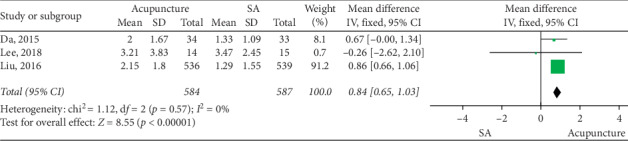
Forest plot for CSBM (acupuncture vs SA).

**Figure 4:**
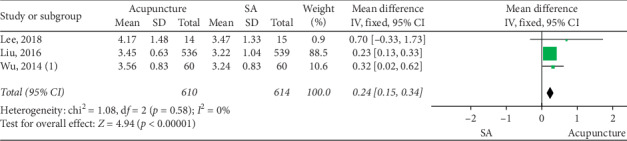
Forest plot for BSFS (acupuncture vs SA).

**Figure 5:**
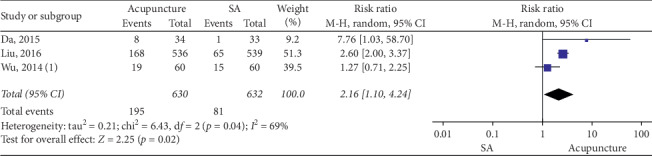
Forest plot for responder rate (acupuncture vs SA).

**Figure 6:**
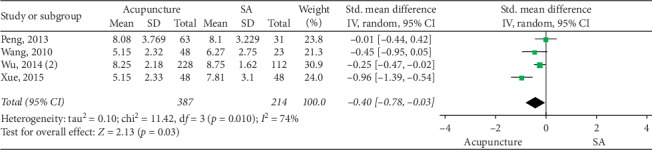
Forest plot for CSS (acupuncture vs SA).

**Figure 7:**
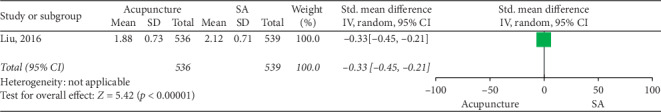
Forest plot for PAC-QOL (acupuncture vs SA).

**Figure 8:**
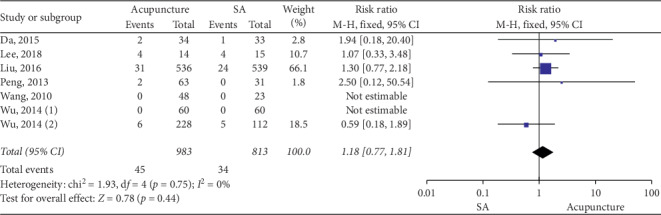
Forest plot for safety evaluation (acupuncture vs SA).

The merged data indicated that the acupuncture group exhibited significantly greater efficacy than the SA group in increasing CSBMs (*p* < 0.00001; MD = 0.84 [95% CI, 0.65 to 1.03]; *I*^2^ = 0%) and improving stool formation (*p* < 0.00001; MD = 0.24 [95% CI, 0.15 to 0.34]; *I*^2^ = 0%), responder rates (*p*=0.02; RR = 2.16; [95% CI, 1.1 to 4.24]; *I*^2^ = 69%), constipation symptoms (*p*=0.03; SMD = −0.4 [95% CI, −0.78 to −0.03]; *I*^2^ = 74%), and the quality of life (*p* < 0.00001; *N* = 1, SMD = −0.33 [95% CI, −0.45 to −0.21]). No increased risk of adverse events was observed (*p*=0.44; RR = 1.18; [95% CI, 0.77 to 1.81]; *I*^2^ = 0%). Sensitivity analysis showed that acupuncture produced a significant decrease in CSS after the removal of one study [30] (*p*=0.02; SMD = −0.23 [95% CI, −0.42 to −0.04]; *I*^2^ = 0%) (Figures 3–8).

### 3.5. Acupuncture vs Medication

**Figure 9:**
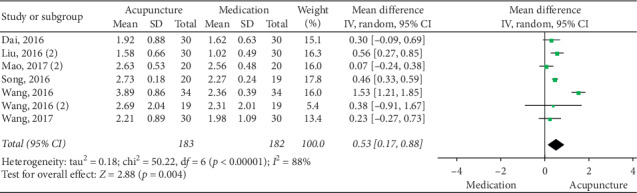
Forest plot for CSBM (acupuncture vs medication).

**Figure 10:**
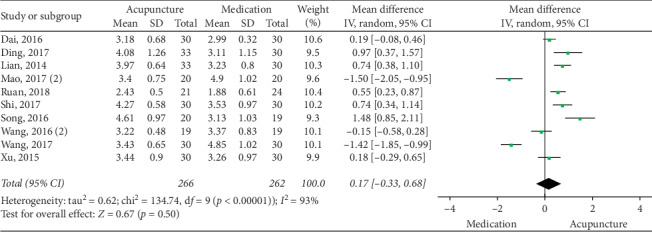
Forest plot for BSFS (acupuncture vs medication).

**Figure 11:**
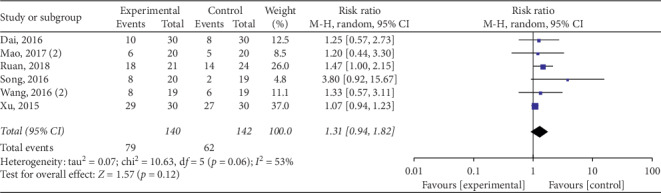
Forest plot for responder rate (acupuncture vs medication).

**Figure 12:**
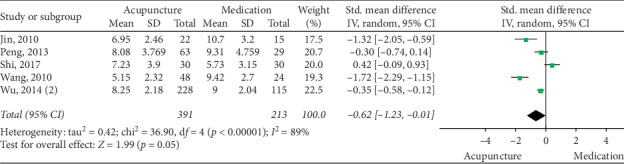
Forest plot for CSS (acupuncture vs medication).

**Figure 13:**
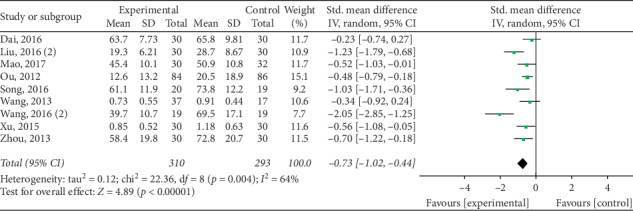
Forest plot for PAC-QOL (acupuncture vs medication).

**Figure 14:**
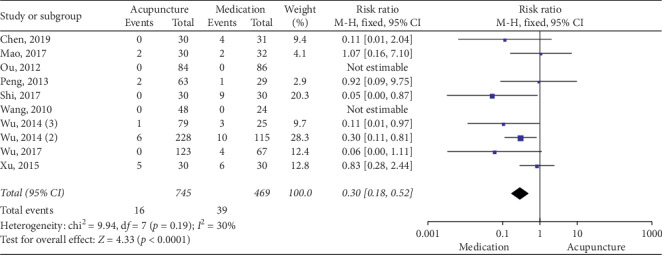
Forest plot for safety evaluation (acupuncture vs medication).

The pooled data indicated that acupuncture was more effective in increasing CSBMs (*p*=0.004; MD = 0.53 [95% CI, 0.17 to 0.88]; *I*^2^ = 88%) and improving patients' quality of life (*p* < 0.00001; SMD = −0.73 [95% CI, −1.02 to −0.44]; *I*^2^ = 64%) than the medication groups. However, there were no significant differences in responder rate (*p*=0.12; RR = 1.31; [95% CI, 0.94 to 1.82]; *I*^2^ = 53%), BSFS (*p*=0.5; MD = 0.17 [95% CI, −0.33 to 0.68]; *I*^2^ = 93%), and CSS (*p*=0.05; SMD = −0.62 [95% CI, −1.23 to −0.01]; *I*^2^ = 89%). Acupuncture was safer than medication (*p* < 0.0001; RR = 0.3; [95% CI, 0.18 to 0.52]; *I*^2^ = 30%) (Figures 9–14).

The sensitivity analysis showed that heterogeneities in CSBM (*p* < 0.00001; MD = 0.37 [95% CI, 0.22 to 0.52 ]; *I*^2^ = 27%), PAC-QOL (*p* < 0.00001; SMD = −0.6 [95% CI, −0.82 to −0.39]; *I*^2^ = 31%), and responder rate (*p*=0.01; RR = 1.45; [95% CI, 1.08 to 1.95]; *I*^2^ = 0%) were reduced significantly after the removal of 1 RCT [36, 43, 53]. However, we did not find a clear source of heterogeneity for CSS and BSFS with an *I*^2^ statistic that ranged from 80% to 93% in subgroup analyses, such as different acupuncture stimulation parameters, different drug groups, age, and disease course.

### 3.6. Subgroup Analysis for Medication

#### 3.6.1. CSBM

**Figure 15:**
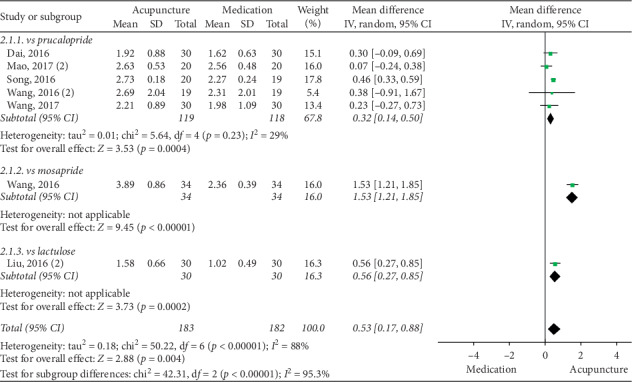
Forest plot for CSBM by subgroup analysis.

Acupuncture had a better effect than prucalopride (*p*=0.0004; WMD = 0.32 [95% CI, 0.14 to 0.5]; *I*^2^ = 29%). However, sensitivity analysis found no significant difference between acupuncture and prucalopride after the removal of one study (*p*=0.1; WMD = 0.18 [95% CI, −0.04 to 0.4]; *I*^2^ = 0%). Two studies showed that acupuncture had a better performance than mosapride and lactulose (Figure 15).

#### 3.6.2. BSFS

**Figure 16:**
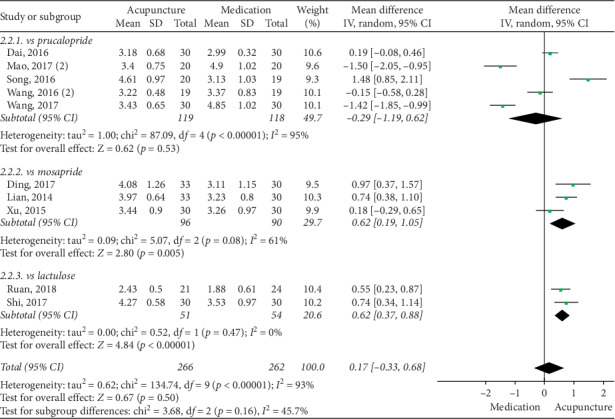
Forest plot for BSFS by subgroup analysis.

Subgroup analysis showed a significant increase in the acupuncture groups' performance on BSFS relative to the lactulose group (*p* < 0.00001; WMD = 0.62 [95% CI, 0.37 to 0.88]; *I*^2^ = 0%) and the mosapride group (*p*=0.005; WMD = 0.62 [95% CI, 0.19 to 1.05]; *I*^2^ = 61%). Acupuncture was not significantly different than the highly heterogeneous comparison with prucalopride (*p*=0.53; WMD = −0.29 [95% CI, −1.19 to 0.62]; *I*^2^ = 95%) (Figure 16).

#### 3.6.3. CSS

**Figure 17:**
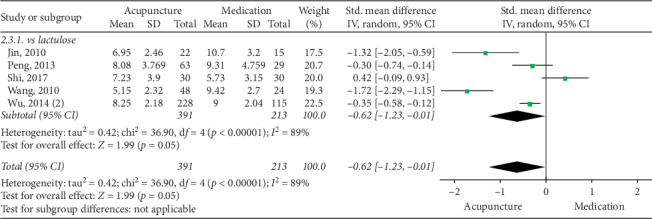
Forest plot for CSS by subgroup analysis.

There was no evidence of a benefit in reducing CSS in the acupuncture group compared to the lactulose group (*p*=0.05; SMD = −0.62 [95% CI, −1.23 to −0.01]; *I*^2^ = 89%). However, sensitivity analysis found that acupuncture was superior to lactulose in reducing CSS after the removal of one study [48] (*p*=0.008; SMD = −0.87 [95% CI, −1.52 to −0.23]; *I*^2^ = 88%) (Figure 17).

#### 3.6.4. PAC-QOL

**Figure 18:**
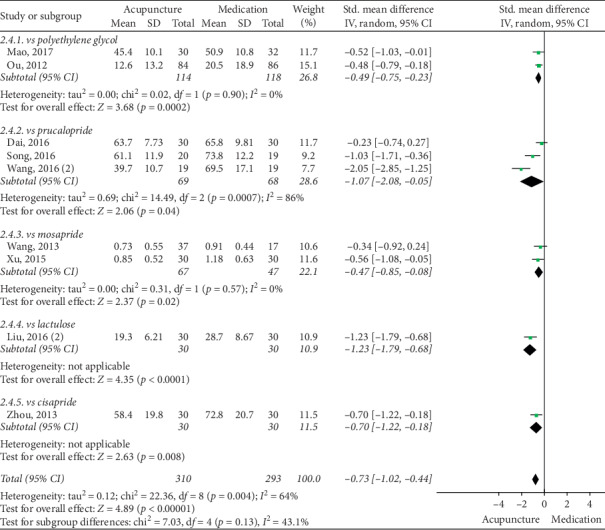
Forest plot for PAC-QOL by subgroup analysis.

Subgroup analysis revealed that acupuncture produced a significant benefit compared with polyethylene glycol (*p*=0.0002; SMD = −0.49 [95% CI, −0.75 to −0.23]; *I*^2^ = 0%) and mosapride (*p*=0.02; SMD = −0.47 [95% CI, −0.85 to −0.08]; *I*^2^ = 0%). Two studies reported that the acupuncture group had a lower score than the cisapride group (*p*=0.008, *N* = 1, *n* = 60, 95% CI, −1.22 to −0.18) and lactulose group (*p* < 0.0001, *N* = 1, *n* = 60, 95% CI, −1.79 to −0.68). However, high heterogeneity was found in comparisons with prucalopride (*p*=0.04; SMD = −1.07 [95% CI, −2.08 to −0.05]; *I*^2^ = 86%) (Figure 18).

#### 3.6.5. Responder Rate

**Figure 19:**
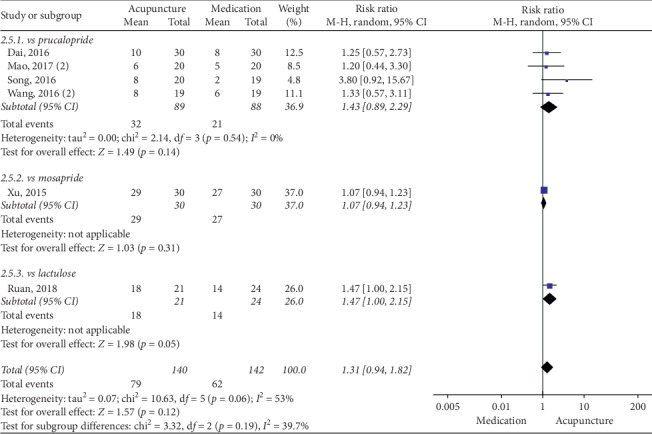
Forest plot for responder rate by subgroup analysis.

Prucalopride (*p*=0.07; RR = 1.25; [95% CI, 0.98 to 1.6]; *I*^2^ = 14%), mosapride (*N* = 1, *n* = 60, *p*=0.31; [95% CI, 0.94 to 1.23]), and lactulose (*N* = 1, *n* = 45, *p*=0.05; [95% CI, 1 to 2.15]) failed to achieve statistical significance (Figure 19).

#### 3.6.6. Safety Evaluation

**Figure 20:**
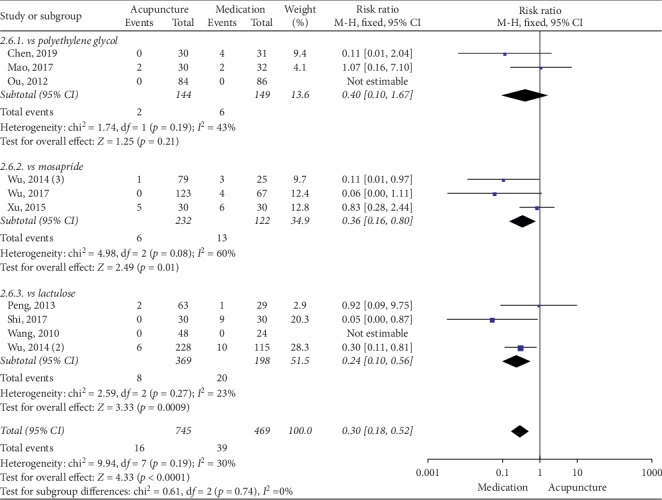
Forest plot for safety evaluation by subgroup analysis.

The subgroup analysis suggested that acupuncture produced no significant difference compared with polyethylene glycol (*p*=0.21; RR = 0.4; [95% CI, 0.1 to 1.67]; *I*^2^ = 43%). Methodologically, acupuncture was safer than lactulose (*p*=0.0009; RR = 0.24; [95% CI, 0.1 to 0.56]; *I*^2^ = 23%) and mosapride (*p*=0.01; RR = 0.36; [95% CI, 0.16 to 0.8]; *I*^2^ = 60%) (Figure 20).

### 3.7. GRADE Evaluation

We only evaluated the qualities of the outcomes that compared acupuncture with SA, and the quality of that evidence ranged from very low to moderate (Table 3). The major reasons for downgrading the evidence quality were inconsistency and reporting bias. The levels of evidence quality were moderate for PAC-QOL and safety evaluation, low for CSBM, BSFS, and responder rate, and very low for CSS.

## 4. Discussion

### 4.1. Principal Results

The present review examined 28 RCTs involving 3525 participants that studied the effects of acupuncture treatment on the management of FC. Acupuncture was associated with the magnitude of clinically relevant effects in reducing the severity of FC compared with SA and pharmacological treatments (polyethylene glycol, prucalopride, mosapride, cisapride, and lactulose). With regard to SA comparison, acupuncture treatment may not increase the risk of adverse events and may be more efficient in increasing CSBMs, improving stool formation, alleviating constipation symptoms, and promoting the quality of life and responder rates. This study found that SA was inferior to real acupuncture for patients, which was consistent with previous findings [18, 56, 57]. However, the evidence quality was relatively low because of inconsistency and reporting bias. Our meta-analysis showed that acupuncture may be more effective than pharmacological treatment in increasing weekly CSBMs and improving the quality of life and responder rate. The data suggested that acupuncture caused fewer adverse events. However, no significant benefits in stool formation or clinical symptoms of FC were found in patients who received acupuncture compared with drug with high heterogeneity.

Previous studies showed that many factors influenced the efficacy of acupuncture, such as age, comorbidity, gender, disease severity, stimulation of acupuncture, expectations of patients, and doctor-patient interaction, which may be sources of heterogeneity [58–60]. However, due to the inability to obtain more relevant data, we cannot analyze based on relevant influencing factors. The present study only found that the heterogeneity may be caused by different control group. There were two outcomes (CSS and BSFS) without an apparent source of heterogeneity compared between acupuncture and medication. Our careful data analysis suggested that small sample size, the specificity of outcome indicators, and statistical methods may be the reasons for heterogeneity. For example, different types of variables, such as considering the BSFS as a continuous or categorical variable, may have differentially influenced the heterogeneity. However, most of the results of the included high-quality studies did not include categorical variable data, and we cannot judge whether the two analysis methods have different effects on the results.

The current study included five Western medicines that were directly compared with acupuncture, including saline laxatives (polyethylene glycol), osmotic laxatives (lactulose), and 5‐HT agonists (prucalopride, mosapride, and cisapride). The guidelines have different mechanisms of action and side effects, such as mosapride, which only acts in the upper digestive tract, and cisapride, which is associated with cardiac arrest [61, 62]. Therefore, to avoid the effect of different mechanisms of action and side effects of drugs on the results, we added a different subgroup analysis based on drug control.

Compared with the first-line agents, the subgroup analysis showed that acupuncture may be more effective than lactulose in increasing weekly CSBMs and more advantageous than polyethylene glycol, prucalopride, and lactulose in improving the quality of life. It was suggested that acupuncture caused fewer adverse events than polyethylene glycol and lactulose. However, the evidence is insufficient because of the drug characteristics, small sample size, and inadequate blinding. Studies showed that polyethylene glycol and lactulose were not effective in alleviating abdominal pain and bloating, which directly affect the quality of life of patients [62]. Because of the inert characteristics of acupuncture, it is difficult to implement a blinded method when choosing medication as a control. Therefore, the effectiveness of acupuncture is impossible to exclude because the patient has greater expectations for acupuncture treatment, especially improvements in subjective feelings.

### 4.2. Strengths

This meta-analysis has several strengths. Compared with previous reviews and meta-analyses, the unified specifications of the FC diagnostic criteria for inclusion in this review were all Rome III, except for one RCT [44]. We included several high-quality multicenter RCTs with large sample sizes from 2010 to 2019, including the largest trial with 1075 patients, which pinpointed that EA reduced the scores of constipation symptoms and quality of life in patients with chronic severe functional constipation after 8 weeks [28]. This review observed more comprehensive outcome indicators related to the effectiveness of FC treatment involving the frequency and symptoms of defecation, stool form, quality of life, and side effects and compared acupuncture with other clinical drugs for FC to show the effectiveness and safety of acupuncture more intuitively.

### 4.3. Limitations and Implications for Research and Practice

There are some limitations in this study. First, blinding remains a common challenge in acupuncture clinical research, and 19 RCTs had a high risk in the blinding of participants and personnel in our risk of bias assessment. Future trials should strengthen the effectiveness of the blinding method and adopt appropriate fake devices to examine research questions, minimize potential bias, and improve the quality of the evidence. Second, most RCTs were performed in China, which may lead to publication bias and affect the validity and reliability of this systematic review. Databases in other languages should be considered for inclusion in the future, such as Japanese, Korean, and German.

There are still some unanswered questions. First, the optimal variables deserve further investigation, including acupuncture type, frequency, duration, and selection of acupoints in acupuncture treatment. Our literature review found that many other types of acupuncture are used to treat FC, including warm needles, acupoint injections, and ear needles. No research showed that acupuncture or EA was the best method to treat FC, which requires further research.

Second, recent studies investigated the effectiveness of acupuncture for chronic severe FC, but there was no comprehensive data analysis to determine the efficacy of acupuncture for chronic severe FC. There remain further unanswered questions about which patients may find acupuncture most beneficial in terms of FC severity. We know that patients generally experience a range of other symptoms during constipation, such as anxiety, abdominal pain, and anorexia. Traditional acupuncturists consider these symptoms when making treatment plans. More trials of this type are needed to model real-world settings.

Finally, our subgroup analysis results showed that comparisons of acupuncture and drugs revealed many uncertainties in outcome indicators. The most prominent requirement in the past was to perform more high-quality RCTs to evaluate the effectiveness of acupuncture for the treatment of FC. This meta-analysis suggested that acupuncture was better than some clinical medicines in increasing defecation frequency and quality of life. Therefore, more trials are needed in the future to clarify the clinical advantages and disadvantages of acupuncture and explore how acupuncture can supplement or replace the shortage of existing drugs.

## 5. Conclusions

This systematic review suggests that acupuncture for FC is safe and effective, especially in terms of increased stool frequency and improved constipation symptoms, stool formation, and quality of life, but the relationship between acupuncture and drugs is not clear. In the future, high-quality RCTs are still needed to provide evidence to support these conclusions and examine the alternative or complementary relationship between acupuncture and existing drugs for the treatment of FC.

## Figures and Tables

**Table 1:** The search strategy in PubMed.

Number	Search items
1	Functional constipation
2	Chronic functional constipation
3	Chronic constipation
4	Idiopathic constipation
5	Slow transit constipation
6	Functional gastrointestinal disorder
7	Functional defecatory disorder
8	Chronic severe functional constipation
9	Constipation
10	FC
11	CC
12	CSFC
13	Or 1–12
14	Acupuncture
15	Acupuncture therapy
16	Acupuncture needle
17	Manual acupuncture
18	Electroacupuncture
19	Needling
20	MA
21	EA
22	Or 14–21
23	Randomized controlled trial
24	Controlled clinical trial
25	Randomized
26	Randomly
27	Trial
28	Or 23–27
29	Exp animals/not humans
30	28 not 29
31	13 and 22 and 30

**Table 2:** Characteristics of included studies.

Author	Study sites	*n*	Diagnostic criteria	Participants	Participants' age (years, M ± SD)	Disease course (M ± SD)	Duration	Outcomes
*Acupuncture vs sham electroacupuncture*								
Da et al. [26]	1	67	Rome III	Treatment: 34	37.94 ± 18.06	139.59 ± 112.68 mos	8 weeks	①③⑥
				Control: 33	37.00 ± 17.89	106.21 ± 91.98 mos		
Lee et al. [27]	1	29	Rome III	Treatment: 14	49.6 ± 12.7	Not reported	4 weeks	①②⑥
				Control: 15	50.0 ± 10.5	Not reported		
Liu et al. [28]	15	1075	Rome III	Treatment: 536	47.01 ± 16.5	130.8 ± 122.6 mos	8 weeks	①②③⑤⑥
				Control: 539	47.33 ± 15.8	132.7 ± 127.0 mos		
Wu [29]	1	120	Rome III	Treatment: 60	49 ± 34.5	68.5 ± 94.5 mos	8 weeks	②③⑥
				Control: 60	52.63 ± 12.9	101 ± 102.2 mos		
Xue et al. [30]	1	96	Rome III	Treatment: 48	48.85 ± 13.30	7.65 ± 6.48 yrs	4 weeks	④⑥
				Control: 48	45.25 ± 11.28	8.48 ± 5.76 yrs		

*Acupuncture vs polyethylene glycol*								
Chen [31]	1	61	Rome III	Treatment: 30	48.80 ± 8.18	5.06 ± 3.66 mos	4 weeks	⑥
				Control: 31	48.58 ± 8.14	4.94 ± 3.68 mos		
Mao [32]	1	62	Rome III	Treatment: 30	74.5	1 mos	2 weeks	⑤⑥
				Control: 32	73	1 mos		
Ou [33]	1	170	Rome III	Treatment: 84	48.03 ± 17.19	24.52 ± 11.32 mos	4 weeks	⑤⑥
				Control: 86	46.64 ± 15.71	23.5 ± 10.36 mos		

*Acupuncture vs mosapride*								
Ding et al. [34]	1	63	Rome III	Treatment: 33	34.83 ± 11.76	5.71 ± 2.54 yrs	4 weeks	②
				Control: 30				
Lian et al. [35]	1	63	Rome III	Treatment: 33	26.85 ± 8.27	3.44 ± 2.56 yrs	4 weeks	②
				Control: 30	27.60 ± 7.86	2.92 ± 2.24 yrs		
Wang et al. [36]	1	68	Rome III	Treatment: 34	47.8 ± 10.1	7. 6 ± 6.4 yrs	4 weeks	①
				Control: 34	46. 6 ± 11. 0	8.1 ± 5.9 yrs		
Wang [37]	1	54	Rome III	Treatment: 37	28.08 ± 13.42	95.43 ± 103.03 mos	4 weeks	⑤
				Control: 17	27.59 ± 9.70	92.00 ± 78.48 mos		

*Acupuncture vs prucalopride*								
Dai [38]	1	60	Rome III	Treatment: 30	40.48 ± 2.96	110.76 ± 17.4 mos	8 weeks	①②③⑤
				Control: 30	42.80 ± 3.92	150.48 ± 30.84 mos		
Mao [39, 40]	1	56	Rome III	Treatment: 28	44.85 ± 7.71	3.78 ± 2.12 yrs	8 weeks	①②③
				Control: 28	46.95 ± 9.83	3.88 ± 2.36 yrs		
Song [41]	1	39	Rome III	Treatment: 20	51.40 ± 12.90	Not reported	8 weeks	①②③⑤
				Control: 19	49.16 ± 12.31	Not reported		
Wang et al. [42]	1	60	Rome III	Treatment: 30	46 ± 7	4.52 ± 2.36 yrs	4 weeks	①②
				Control: 30	47 ± 8	4.64 ± 2.65 yrs		
Wang [43]	1	38	Rome III	Treatment: 19	41.53 ± 16.15	76.68 ± 7.75 mos	8 weeks	①②③⑤
				Control: 19	35.29 ± 13.26	76 ± 4.93 mos		

*Acupuncture vs cisapride*								
Zhou et al. [44]	1	60	The guidelines for clinical research	Treatment: 30	37. 36 ± 10. 32	2. 54 ± 1. 63 yrs	4 weeks	⑤
				Control: 30	39. 58 ± 11. 63	2. 72 ± 1. 76 yrs		
*Acupuncture vs lactulose*								
Jin [45]	1	37	Rome III	Treatment: 22	39.14 ± 14.45	115.18 ± 108.08 mos	4 weeks	④
				Control: 15	45.13 ± 17.09	157.4 ± 142.24 mos		
Liu et al. [46]	1	60	Rome III	Treatment: 30	53. 13 ± 9. 65	3.70 ± 2. 54 yrs	2 weeks	①⑤
				Control: 30	52.76 ± 8.87	3.96 ± 2.68 yrs		
Ruan et al. [47]	1	45	Rome III	Treatment: 21	68 ± 9	17.90 ± 9.77 mos	3 weeks	②③
				Control: 24	69 ± 8	16.92 ± 10.04 mos		
Shi [48]	1	60	Rome III	Treatment: 30	64.87 ± 4.208	5.27 ± 3.51 yrs	4 weeks	②④⑥
				Control: 30	66.27 ± 3.513	5.5 ± 3.94 yrs		

*Acupuncture vs sham acupuncture vs lactulose*								
Peng et al. [49, 50]	3	128	Rome III	Treatment: 64	53 ± 13	125.1 ± 128.6 mos	4 weeks	④⑥
				Control A: 33	52 ± 17	118 ± 105.8 mos		
				Control B: 31	59 ± 12	97.8 ± 123 mos		
Wang et al. [51]	1	95	Rome III	Treatment: 48	48.8 ± 13.3	7.65 ± 6.48 yrs	4 weeks	④⑥
				Control A: 24	40.8 ± 10.0	9.46 ± 5.89 yrs		
				Control B: 23	44.6 ± 15.2	7.65 ± 5.65 yrs		
Wu et al. [52]	5	475	Rome III	Treatment: 228	45.88 ± 16.85	110.84 ± 99.85 mos	4 weeks	④⑥
				Control A: 112	46.25 ± 16.81	109.25 ± 100.70 mos		
				Control B: 115	44.12 ± 17.48	111.04 ± 110.15 mos		

*Acupuncture vs mosapride vs mosapride & sham electroacupuncture*								
Xu [53]	1	90	Rome III	Treatment: 30	35.26 ± 19.07	8.88 yrs	4 weeks	②③⑤⑥
				Control A: 30	35.42 ± 15.28	8.71 yrs		
				Control B: 30	36.00 ± 17.20	8.83 yrs		

*Low intensity acupuncture vs high intensity acupuncture vs mosapride*								
Wu et al. [54]	3	190	Rome III	Treatment A: 58	34.00 ± 15.62	70.44 ± 85.53 mos	4 weeks	⑥
				Treatment B: 65	37.20 ± 18.19	86.29 ± 104.06 mos		
				Control: 67	43.60 ± 17.90	68.09 ± 74.13 mos		

*Shu-mu vs He vs Shu-mu-he vs mosapride*								
Wu et al. [55]	1	104	Rome III	Treatment A: 19	61 (16)	130 mos	4 weeks	⑥
				Treatment B: 34	53 ± 12	123 mos		
				Treatment C: 26	56 ± 9	217.35 mos		
				Control: 25	55 ± 11	130 mos		

Notes: M ± SD, the mean ± standard deviation; mos, months; yrs, years; ① complete spontaneous bowel movement (CSBM); ② Bristol Stool Form Scale (BSFS); ③ responder rate; ④ constipation symptoms scores (CSS); ⑤ Patient Assessment Of Constipation Quality Of Life (PAC-QOL) questionnaire; ⑥ safety evaluation.

**Table 3:** GRADE evaluation: acupuncture compared to sham acupuncture.

Condition	No. of participants (studies)	Design	Limitations	Inconsistency	Indirectness	Imprecision	Other considerations	MD or SMD or RR (95% CI)	Quality
CSBM	1171 (3)	RCT	No serious	Serious	No serious	No serious	Reporting bias	0.84 (0.65 to 1.03)	Low
BSFS	1224 (3)	RCT	No serious	Serious	No serious	No serious	Reporting bias	0.24 (0.15 to 0.34)	Low
CSS	432 (4)	RCT	Serious	Serious	Serious	Serious	Reporting bias	−0.42 (−0.81 to −0.02)	Very low
PAC-QOL	1075 (1)	RCT	No serious	No serious	No serious	No serious	Reporting bias	−0.33 (−0.45 to −0.21)	Moderate
Responder rate	1262 (3)	RCT	No serious	Serious	No serious	No serious	Reporting bias	2.16 (1.1 to 4.24)	Low
Safety evaluation	1627 (7)	RCT	Serious	No serious	No serious	No serious	None	1.21 (0.78 to 1.87)	Moderate

RCT, randomized controlled trial; MD, mean difference; SMD, standard mean difference; RR, relative risk; CI, confidence interval.

## Supplementary Materials

Supplementary Materials S1: checklist of items to include when reporting a systematic review or meta-analysis.
